# CHARACTERIZATION OF BY-PRODUCTS WITH HIGH FAT CONTENT DERIVED FROM THE PRODUCTION OF BOVINE GELATIN

**DOI:** 10.12688/f1000research.128622.1

**Published:** 2022-12-23

**Authors:** Victor Alonso Garcia Londoño, Natalia Marín González, Diego Fernando Roa-Acosta, Lina Marcela Agudelo Laverde, Laura Botero, Liliana Maria Lellesch

**Affiliations:** 1 Departamento de Química Orgánica FCEN, Universidad de Buenos Aires, Ciudad Autónoma de Buenos Aires, Buenos Aires, 1428, Argentina; 2 Instituto de Tecnología en Polímeros y Nanotecnología, UBA-CONICET, Ciudad Autónoma de Buenos Aires, Buenos Aires, 1128, Argentina; 3 Laboratorio de Investigación, Desarrollo e Innovación, PROGEL S.A.S, Manizales, Caldas, 170001, Colombia; 4 Departamento de Agroindustria, Universidad del Cauca, Popayán, Cauca, 190001, Colombia; 5 Programa de Ingeniería de Alimentos, Universidad del Quindío, Armenia, Quíndio, 630001, Colombia

**Keywords:** fats, gelatin, by-products, characterization, circular economy

## Abstract

**Background:** Gelatin is a protein obtained by partial hydrolysis of collagen contained in skins, connective tissue and/or animal bones, which are by-products of the meat industry. The main raw material to produce bovine gelatin is the dermis of the skin, but there is a variation in fat and moisture content depending on the bovine skin origin. As a contribution to the circular economy and sustainability, these by-products with high fat content and the fat released from them during the gelatin production process can be managed for food industries, mainly in the development or formulation of animal feed.

**Methods:** For the initial physicochemical characterization, moisture, fat, protein and ashes content were determined. Once the by-products with high fat content were identified, alteration parameters such as acidity, peroxide and saponification indexes were evaluated. Additionally, thermal, rheological and fatty acid composition characterization was carried out in order to study the possible applications of the by-products.

**Results and Discussion:** The results showed that some of the by-products presented fat content values lower than 15.0%, so the viability of their use is limited. On the other hand, some by-products have more than 30% fat content, however, they can only be removed manually, and the efficiency of this process is low. By-products removed from the supernatant in the extractors presented 99.9 and 98.9% of fat, and there is the possibility of conditioning a mechanical method for their extraction. The determination of alteration and oxidation parameters, thermal and rheological characterization, fatty acid profile and solid fat content were carried out only on these by-products. According to the characterization, these by-products could be valued and used in the formulation of animal feed, however, they present some limitations for some applications such as biodiesel production or food industry.

## Introduction

The gelatin production process consists of three main steps: preparation, processing and mixing. One of the raw materials used is the dermis of bovine leather, which is a by-product of the meat industry. The dermis contains collagen (molecule of interest in the gelatin production process) within its native structure. The preparation step aims to prepare the raw material so that it can be later processed in the production area. It begins with the reception of the cattle hides, which are subjected to basic and acid treatments that favor the extraction of the collagen. During the processing step, the extraction and concentration of the collagen are carried out. The concentrated liquor goes through a high temperature treatment (sterilization) to eliminate any possible microorganisms present. The sterile liquor is then chilled and extruded (in cold) as noodles, and the gelled material is deposited as a bed onto an endless, open weave, stainless steel belt, and is subsequently dehydrated in a drying tunnel. Finally, the dried gelatin is crushed and ground. In the mixing step, the ground gelatin is physiochemically analyzed and mixed according to the requirements of each client. In the gelatin production process, five different bovine raw materials can be used: dry, whole, leather, selvedge and trimmings (the one with the highest percentage of fat). The trimmings correspond to different parts of the animal which include udders, gonads, prepuces, and faces. They can be of national origin (CPFC) or imported (PPSL).

In particular, the production of gelatin takes advantage of by-products generated in the leather and meat industry. In the literature review, no studies were found on the use of by-products generated in the gelatin production process. The non-use or under-use of by-products not only leads to the loss of potential income, but also represents an increase in the cost of elimination or disposal of this waste. ^1^ Traditional uses for protein-rich byproducts include food, pet food, livestock feed, and fertilizers. Regarding fatty by-products of animal origin, in recent years, different alternatives have been proposed for their use and recovery: chemical transformation into soaps (derived from fatty acids), formulation of products for the food industry or reincorporation into the food chain as balanced feed for animals, after physical and chemical treatment. ^2^

In recent years, new alternative uses of fat as energy/fuel sources have been developed, such as the production of biodiesel. ^3^ Any raw material that contains triacyl glycerides can be used to produce biofuels: sunflower oil, rapeseed oil, soybean oil, used frying oils, lard, beef tallow. Although in some countries, the production of biofuels comes mainly from oils extracted from oilseeds, especially sunflower (in Spain and Italy) and rapeseed (in Central European countries), the use of waste from used vegetable oils and animal fats has been popularized. ^4^^–^^6^ Different products have been formulated from beef fat for the food industry, among which are substitutes for cocoa butter, ^7^ fat base for the production of margarine with plastic characteristics, ^8^ shortenings ^9^ and products for baking industry. ^6^ In some cases, the fatty acid composition of the by-product is similar to the original product, but in other cases, great variability can be found. These differences can extend to other factors or properties that have incidence on nutritional value, stability or general quality. ^10^

The characterization of the by-products is fundamental since, based on certain results, it is possible to predict, for example, the fat behavior during its processing to obtain a biofuel or its possible applications in the food industry. Among the parameters commonly evaluated are the acidity, iodine and saponification indexes, humidity, fatty acid profile and triacyl glycerides content. ^5^ Another important aspect when characterizing these by-products is to evaluate possible alterations. A typical alteration reaction is the autoxidation of lipids, which is one of the main causes of the deterioration of foods rich in fat, giving rise to the appearance of unpleasant odors and flavors. This reaction occurs primarily with unsaturated fatty acids through a series of free radical chain reactions. Another deterioration reaction that occurs is lipolysis, which is produced from the hydrolysis of lipid ester bonds (enzymatic by the action of lipases or by heat in the presence of water). In animal fats, lipolysis releases fatty acids, some of which (short-chain ones) are responsible for off-flavors and off-odors. Some of the most frequently used chemical indices to determine the development of oxidation are the peroxide index (PI), which measures the amount of peroxides formed; the thiobarbituric acid reactive substances (TBARS), which quantify the secondary products of lipid oxidation; and the acidity index that determines the degree of hydrolysis of an oil or fat. ^10^

Understanding the functions and properties of fats and oil bases is essential for the design of possible applications and uses, or to obtain food products with the desired final attributes. The satisfactory performance of a fat depends on important elements that determine its applicability: the stability during the post-processing period, the total compatibility of the fat with the product for which it is intended and its physical and functional characteristics, such as plasticity and spreadability. ^11^ Therefore, an application study should be based primarily on understanding the relationships among parameters such as fatty acid and triacylglyceride composition, melting point, solid fat content, thermal behavior, crystallization, microstructure and rheological properties. ^12^

The objectives of the present study were to carry out the physicochemical characterization of the by-products generated in the gelatin production process, to analyze oxidation and alteration parameters, thermal behavior, rheological profile, fatty acid profile and solid fat content of the potentially usable fatty by-products.

## Methods

### Sampling

**Figure 1.**
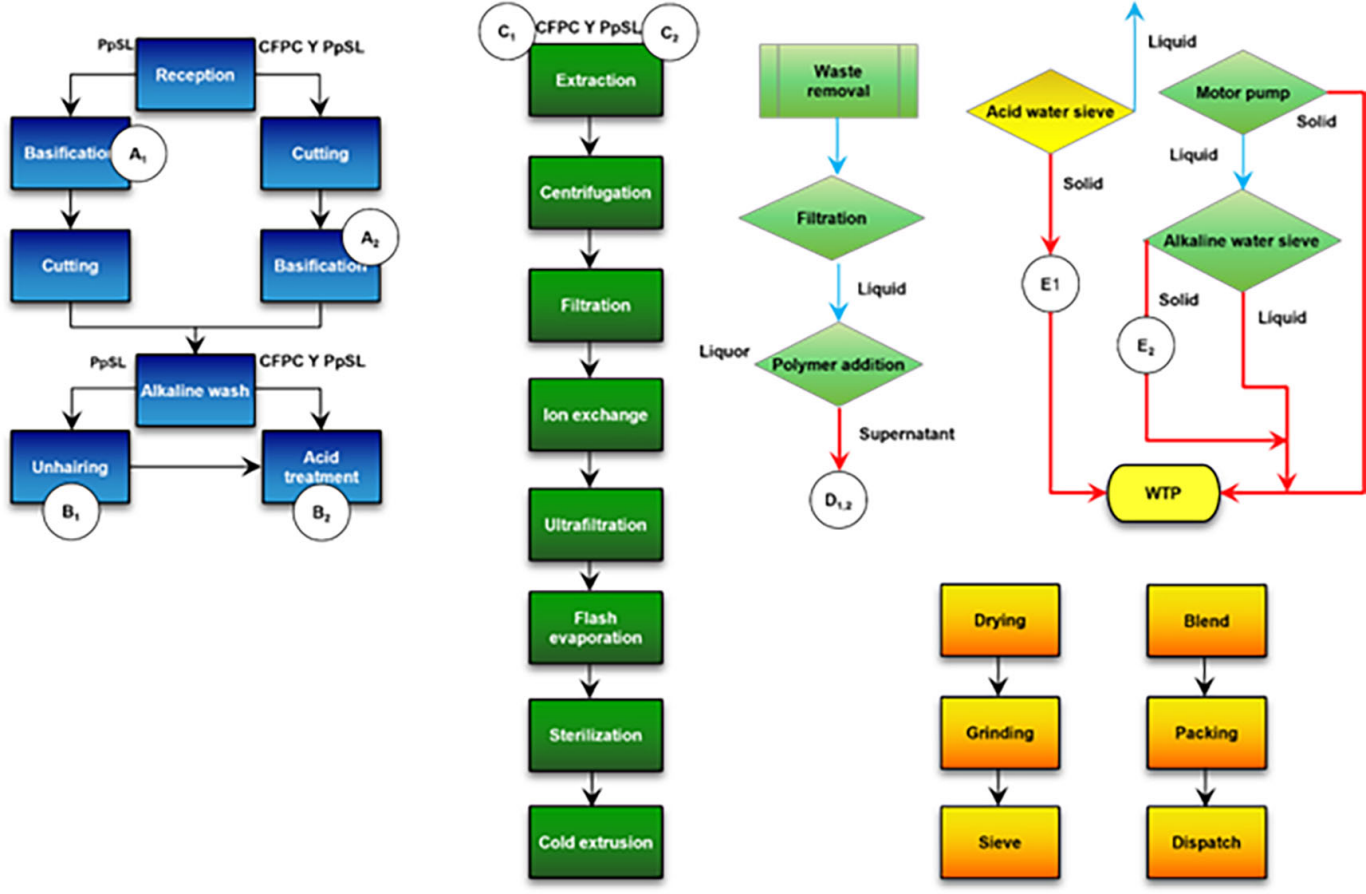
Flow chart and sampling points for fatty by-products.

The sampling of the by-products was carried out in the preparation, processing and wastewater treatment plant (WTP) areas. A total of 10 points were sampled, differentiating by raw material in those points where CFPC and PPSL were treated independently (basification, alkaline wash, and extraction). Figure 1 shows the flow chart and the sampling points.

Sampling was carried out for three consecutive days (29/08/2022 to 31/08/2022) and each sample was analyzed in triplicate in all tests.

### Physicochemical identification of potentially usable by-products

***Moisture content determination***

Moisture content was determined by the indirect method AOAC (Association of Official Analytical Chemists) 964.22, where 5±0.005 g of sample was weighed and dried at 110°C in a forced air oven until constant weight. Constant weight was achieved after approximately 16 h of drying. Weight loss was considered as moisture content and dry residue was considered as dry matter. The results were expressed as a percentage.

***Fat content determination***

Fat content was determined by the direct organic solvent extraction method, AOAC, 960.39. Due to the high moisture content of the by-products, the measurement was made on the dry matter obtained from the moisture determination. The dehydrated material was quantitatively transferred to a cellulose cartridge, covered with cotton and placed in the Soxhlet extractor. Extraction was carried out with anhydrous ethyl ether for 4 h, heating at a condensation rate of 5 or 6 drops per second. The extract was then quantitatively transferred to an Erlenmeyer flask and the remained solvent was evaporated in a water bath and dried at 100°C for 30 min. Finally, it was cooled and weighed. The results were expressed as percentage of fat on wet basis.

***Protein content determination***

Protein determination was performed by the Kjeldahl-Arnold-Gunning method (AOAC, 928.08), by digesting 0.8±0.01 g of sample in a Kjeldahl digestion tube with 6 g of Na _2_SO _4_, 0.3 g of CuSO _4_ and 12 ml of concentrated H _2_SO _4_. The digestion was carried out in a Selecta digester (PRO-NITRO M) under the following conditions: 125°C – 15 minutes; 300°C – 15 minutes; 400°C – 90 minutes. The distillation was carried out in a Selecta automatic Kjeldahl Distiller (Pro-nitro). The digested sample was alkalized and distilled by steam addition. The distillate was collected in 50 ml of saturated boric acid solution with combined indicator. Nitrogen content was determined by titration with 0.1N HCl. Results were expressed as protein percentage using a conversion factor of 6.25.

***Ash content determination***

Based on AOAC 923.03 method, 5±0.01 g of sample was placed in a porcelain capsule of 6 cm of diameter. The sample was first incinerated with a Bunsen burner until complete carbonization and then in a high-temperature muffle furnace at 500°—550°C until constant weight. Finally, it was cooled in a desiccator and weighed. Results were expressed as ash percentage.

### Characterization of potentially usable fatty by-products (alteration and oxidation index, and thermal and rheological characteristics, fatty acid profile and solid fat content)

***Saponification index (SI)***

According to the CTS (Colombian Technical Standard) 335, 2±0.005 g of sample was weighed in an Erlenmeyer flask and 25 mL of ethanolic solution of potassium hydroxide were added together with boiling aids. The reflux condenser was connected to the flask and the solution was allowed to boil slightly, while stirring sporadically. After 60 min of boiling, the condenser was removed and 0.5 mL of phenolphthalein solution was added to the hot solution. It was then titrated with 0.1 N HCl. A reagent blank was analyzed in the same way. The results were expressed as the number of milligrams of KOH required to saponify 1 g of fat.

***Acidity index (AI)***

The acidity index was performed following the procedure established in the CTS 218. A sample of 2.5±0.005 g was weighed in a flask. In a second flask, 50 mL of ethanol containing 0.5 mL of phenolphthalein was heated to boiling. While the temperature was above 70°C, it was neutralized with a 0.1 N KOH solution. The neutralized ethanol was added to the flask with the sample and they were mixed and boiled. Finally, it was titrated with 0.1 N KOH solution. The results were expressed as the number of milligrams of KOH required to neutralize free fatty acids in 1 g of sample.

***Peroxide index (PI)***

According to the Colombian Technical Standard 236, 5.0±0.1 g of sample was weighed and 30 mL of acetic/chloroform solution (3:2) was added. The sample was dissolved by shaking and 0.5 ml of KI solution was added. The flask was covered and stirred. It was kept at rest for 60 s and then 30 mL of water were added. The released iodine was immediately titrated with 0.01 N sodium thiosulfate solution until the solution turned pale-yellow color. Then, 0.5 mL of starch solution was added and the titration was continued until colorless. A reagent blank was also analyzed. The results were expressed in milliequivalents of active oxygen per kg of sample.

***Determination of fatty acid methyl esters (FAMEs)***

For the determination of fatty acid methyl esters, the weighed sample (0.7 g) was placed in a 50 mL three-necked flask with 6 mL of a 0.5 M NaOH solution. This flask was placed at reflux, controlling the temperature (maximum of 60°C), until the sample was completely dissolved. 20 minutes were counted from that moment. Then, the sample was derivatized with 5 mL of BF3 at 14% in MeOH and the reaction was continued for another 20 minutes. Finally, 3 mL of n-heptane was added. After cooling, the derivatized sample was taken to a test tube and 15 mL of saturated NaCl solution was added. An aliquot of 1.5 mL was taken of the organic phase and placed in a vial.

A gas chromatography equipment coupled to a Shimadzu GC-MS QP-2010 mass spectrometer, a system equipped with an AOC-20i autoinjector and an AOC-20s autosampler were used. Chromatographic conditions were: Zebron ZB-5 column 30 m × 0.25 mm I. D, 0.25 μm, temperature ramp programmed from 100°C to 300°C (2 min of heating at 100°C, then the temperature was increased from 7°C/min up to 300°C and held for 5 min). The temperature at the injection port was 250°C, the injection was in split mode (1:20), the column flow was 1.00 ml/min, the total flow was 24.0 ml/min, the temperature at the ion source and at the interface was 290°C. The total analysis time was 36 min.

### Rheological profile

An AR 1500 rheometer (TA Instruments, New Castel, USA) equipped with a Peltier temperature control system in the lower plate and an upper plate of 40 mm diameter and 0° was used. An opening of 1.5 mm was set between the lower and upper plates. The sample was conditioned for 2 min at the measurement temperature before running. A shear stress (Pa) scan was performed as a function of time (5 min) at a shear rate of 50 (s ^-1^). The data were modeled by the Weltman equation, where the parameter A is the instantaneous effort necessary to start the structure de-structuring process during shearing and parameter B indicates the rate of de-structuring of fats. ^13^ The rheological profile was performed at 25°C and 35°C.

$$\text{Effort}=A-B*\mathrm{Ln}\;\left(\text{time}\right)$$

### Thermal behavior

Thermal behavior was determined by differential scanning calorimetry (DSC) with a Netzsch polyma 214 calorimeter. A gaseous N _2_ flow at 50 mL/min was used as carrier gas to avoid humidity in the measurement cell. About 9 mg of each sample was weighed into hermetically sealed 40 μL aluminum pans. The samples were melted at 80°C (heating speed of 10°C/min) for 15 min. Subsequently, cooling to 0°C was carried out at 10°C/min, keeping this temperature for 30 min. Then, the samples were heated up to 20°C (10°C/min) for 30 min and finally, melted at 80°C (heating rate of 5°C/min) for 2 min.

### Solid fat content as a function of temperature

Solid fat content (SFC) of the samples at equilibrium was determined with a Bruker mq 20 Minispec equipment that has a cell with temperature control and a magnetic field of 0.47 Tesla, which operates at a frequency of 20 MHz. A 10 mm diameter glass tube specific for NMR with approximately 6 mL of sample was used. Solid fat content (SFC) was studied following the methodology described in the American Oil Chemists' Society (AOCS Cd 16b 93 for confectionery fats). The samples were prepared in triplicate and the results were expressed by the average of three values and their standard deviation. The experimental protocol was: the sample was melted at 100°C and kept isothermally for 15 min in order to erase its thermal history, then it was cooled to 60°C for 5 min, followed by further cooling to 0°C for 90 min. Tempering was carried out at 26°C for 40±0.5 h. Cooling at 0°C was repeated for 90 min and finally, it was kept for 60 min at each of the selected crystallization temperatures (CT) for the determination of the SFC of the fat from extractors (CFPC and PPSL): 10, 15, 20, 25, 30, 35 and 40°C.

## Results

### Physicochemical identification of potentially usable by-products

The physicochemical parameters of the identified fatty by-products are shown in Table 1.

**Table 1.** Physicochemical parameters of the identified fatty by-products (n.d: not determined).

Area	Sampling location	Raw material	Moisture (%)	Fat (%)	Protein (%)	Ash (%)
Preparation	Basification tanks	CFPC (A _1_)	42.2±0.8	39.8±3.9	6.5±0.9	1.9±0.1
		PPSL (A _2_)	48.2±16	30.7±1.9	3.4±1.2	2.7±0.4
	Alkaline wash	CFPC (B _1_)	74.6±4.4	13.6±1.4	4.9±1.2	4.1±0.4
		PPSL (B _2_)	79.9±4.8	11.7±1.7	3±0.2	4.2±0.1
Processing	Extractors	CFPC (C _1_)	n.d	99.9±0.1	n.d	0.004±0.001
		PPSL (C _2_)	n.d	98.9±0.3	n.d	0.002±0.001
	Polymer waste tank A (D _1_)	----	82.7±0.6	3.5±0.4	8.4±0.6	0.7±0.1
	Polymer waste tank B (D _2_)	----	80.5±0.6	3.7±1	8.4±0.4	0.3±0.1
WTP	Acid water sieve (E _1_)	----	87.4±2.5	7.6±0.7	6.2±0.3	2.4±0.4
	Alkaline water sieve (E _2_)	----	73.0±4.8	12±1	2±0.2	4.3±1

The results showed that some of the by-products (B _1_, B _2_, D _1_, D _2_, E _1_ and E _2_) presented fat content values lower than 15.0%, so the viability of their use is limited. On the other hand, by-products A _1_ and A _2_ have more than 30% fat content, however, they can only be removed manually and the efficiency of this process is low. By-products C _1_ and C _2_ presented 99.9 and 98.9% of fat, respectively, and a very low ash content. These fatty by-products are removed from the supernatant in the extractors and there is the possibility of conditioning a mechanical method for their extraction. Given the above, the determination of alteration and oxidation parameters, thermal and rheological characterization, fatty acid profile and solid fat content as a function of temperature were carried out only on C _1_ and C _2_ by-products.

### Alteration and oxidation parameters of potentially usable by-products (C _1_ and C _2_)

Table 2 shows the saponification, acidity and peroxide indices obtained for the potentially usable fat by-products.

**Table 2.** Alteration and oxidation parameters of potentially usable by-products.

Area	Sampling location	Raw material	SI mg KOH/g	AI mg KOH/g	PI meq O _2_/Kg
Processing	Extractors	CFPC (C _1_)	190±1	11.3±0.6	3.0±0.3
		PPSL (C _2_)	194±5	35.2±15.9	10.5±1.0

Resolution 2154 of 2012 of the Ministry of Health and Social Protection of the Republic of Colombia (chapter X) establishes the physicochemical requirements for food tallow, defined as the product obtained by the fusion of fatty, clean and healthy tissues, including fat from trimmings and related muscles and bones, from bovine (Bos taurus) and/or sheep (Ovis aries) animals. C _1_ and C _2_ saponification index values were within the parameters established in the mentioned resolution (SI between 190 and 202 mg KOH/g), but neither of the two by-products complied with the acidity parameters (AI < 2.5 mg of KOH/g). The peroxide value for C _1_ was within the established limit (<10 meq active oxygen/kg), while C _2_ was just within the established limit. The difference in the oxidation and alteration parameters between the fatty by-products obtained from the two raw materials, CFPC (C _1_) and PPSL (C _2_), was mainly due to the chemical treatments and transport times to which they are subjected before being received at the gelatin factory. CFPC is unhaired in local tanneries where sulfides are generally used. Normally, this material arrives at the gelatin factory between 2 and 4 days after being processed in the tannery. PPSL is a raw material imported principally from Argentina and the United States, and for its conservation during maritime transport, salt or brines are added. Moreover, the minimum time between its obtention and its arrival to the gelatin factory in Colombia is 15 days, time enough to cause oxidation and alteration processes. Although neither of the two by-products complied with the parameters of the resolution 2154, there are some strategies for the reduction of free fatty acids ^14^^,^^15^ that could be evaluated in the future.

### Determination of fatty acid methyl esters (FAMEs) of potentially usable by-products (C _1_ and C _2_)

Table 3 shows the percentage of fatty acids for C _1_, C _2_ and a commercial refined beef tallow.

**Table 3.** Percentage of fatty acid methyl esters of a commercial beef tallow, C _1_ and C _2_.

Retention time	FAME	Name	% Composition		
			Beef tallow	CFPC (C _1_)	PPSL (C _2_)
9.8	C14:0	Myristic acid	3.1	3.7	3.4
12.6	C16:0	Palmitic acid	25.7	27.8	34.1
13.12	C16:1	Palmitoleic acid	2.9	3.7	0.5
16.66	C18:0	Stearic acid	22.0	18.3	21.7
17.37	C18:1	Oleic acid	39.2	37.7	37.9
18.26	C18:2	Linoleic acid	2.3	2.8	0.0
18.93	C18:3	Linolenic acid	0.6	0.0	0.0
21.48	C20:0	Arachidonic acid	0.1	1.1	0.5
22.06	C20:1	Eicosenoic acid	0.4	0.0	0.0
		**Saturated**	51.0	50.9	59.6
		**Insaturated**	46.0	44.2	38.4

The fatty acid profile obtained for C _1_ and C _2_ was similar to that of beef tallow, however, it was shown that C _2_ had a lower percentage of unsaturated fatty acids, possibly due to autoxidation or rancidity processes generated by the previous treatment in tanneries and to the time and conditions of storage and transport. Beef tallow is a raw material normally used in the biodiesel industry, but it has unfavorable properties because of the presence of a high concentration of saturated fatty esters (stearic and palmitic). In fact, it is normally mixed with other raw materials with higher content of unsaturated fatty acids. ^16^ Taking into account that C _2_ has about 9% more saturated fatty acids than standard beef tallow and a high content of free fatty acids, this by-product is not attractive for the biodiesel industry. Some alternatives for the use of these by-products are to obtain fractions with different properties through thermal fractionation ^17^ or chemical or enzymatic esterification. ^18^

### Rheological profile of potentially usable by-products (C _1_ and C _2_)

**Figure 2.**
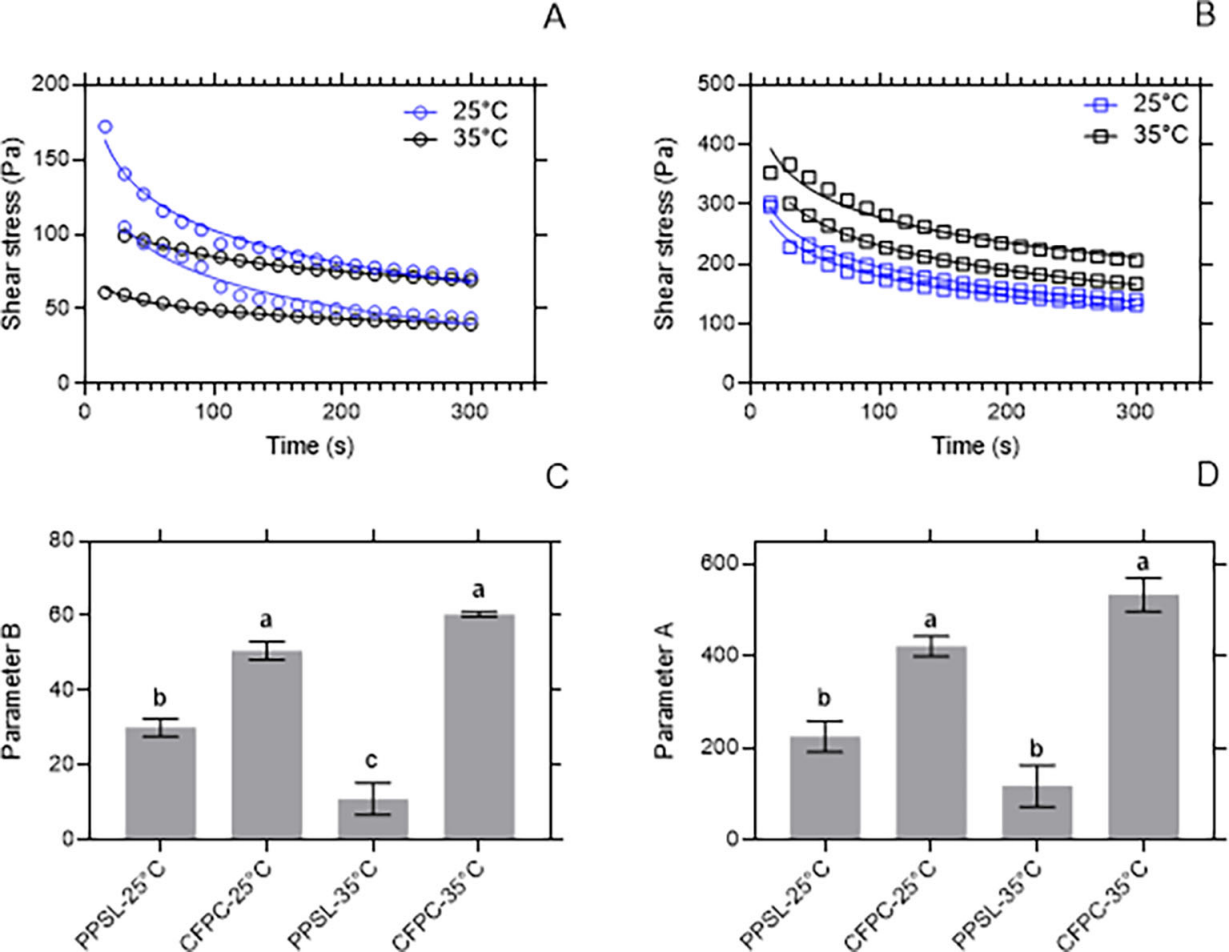
Rheological profile of potentially usable by-products. (A) shows the shear stress behavior for C _2_ subjected to a shear rate of 50 s ^-1^ for 5 minutes at 25°C and 35°C. (B) shows the shear stress behavior for C _1_ subjected to a shear rate of 50 s ^-1^ for 5 minutes at 25°C and 35°C. (C) shows the rate of de-structuring (parameter B) for C _1_ and C _2_ at 25°C and 35°C. (D) shows the initial resistance (parameter A) for C _1_ and C _2_ at 25°C and 35°C.

Figures 2A and 2B show the decrease of the shear stress of C _1_ and C _2_ with time. This type of behavior is characteristic of thixotropy and the shear stress tends to become constant with time until reaching an equilibrium shear stress (σe). The byproduct C _1_ (CFPC) showed σe greater than 150 Pa, while the σe of C _2_ was less than 100 Pa. The parameter B of C _1_ was greater than that of C _2_. This could indicate that C _1_ destructures faster when it applied a shear stress, so it can slide over a surface more easily than C _2_. It is important to note that this ease of sliding on a surface does not change significantly with an increase of 10°C of temperature. Otherwise, parameter B of C _2_ is affected by the temperature change ( Figure 2C). It is also observed that the initial resistance factor (parameter A) was higher in C _1_ and this did not depend significantly on the temperature.

This behavior is influenced by the relationship between saturated and unsaturated fatty acids in each of the samples. By-product C _1_ could be included in the preparation of puff pastry-type bakery products, in which fats that are easily mixed with flour are needed.

### Thermal behavior of potentially usable by-products (C _1_ and C _2_)

**Figure 3.**
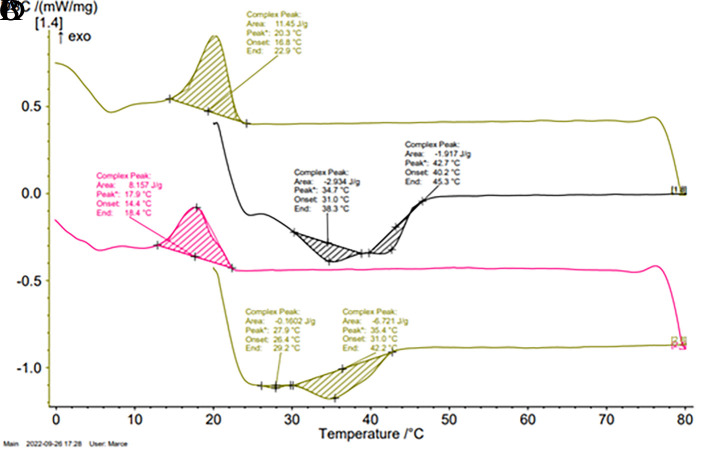
Typical thermograms obtained for by-products C _1_ and C _2_. (A) Shows the crystallization peak of C _1_. (B) Shows the melting peaks of C _1_. (C) Shows the crystallization peak at 20°C of C _2_. (D) Shows the melting peaks of C _2_.

Figures 3A and 3C showed an exothermic peak, attributed to the phenomenon of fat crystallization. In the C _2_ by-product, this phenomenon started at slightly lower temperatures, however, there were no significant differences between the crystallization temperature of the two by-products. Figures 3B and 3D showed two endothermic peaks for each of the by-products analyzed, attributed to the fat melting phenomenon. There was a peak at 35°C in both by-products thermograms, however, in the thermogram of C _2_, there was a peak at 28°C absent in that of C _1_. On the other hand, C _1_ showed a peak at 43°C that was not evidenced in C _2_ thermogram. These results were correlated with the behavior in the solid fat percentage profiles as a function of temperature. The application of a thermal fractionation to the by-product C _1_ would allow obtaining two fractions with different ranges of applicability, which would enhance its value.

**Table 4.** Temperatures and areas of crystallization and melting determined in the by-products C _1_ and C _2_ by DSC.

By-product	Crystallization		Melting			
	Area J/g	Tp °C	Area J/g	Tp °C	Area J/g	Tp °C
**C**_**1**_ **(CFPC)**	10.8±1.1	21.4±1.1 ^a^	1.8±0.2	34.9±0.1 ^c^	3.4±0.7	43±0.3 ^e^
**C**_**2**_ **(PPSL)**	6.9±1.9	18.8±2.7 ^a^	0.3±0.2	28.1±0.5 ^d^	5.7±1.3	35.5±0.2 ^c^

### Solid fat content as a function of temperature (SFC) of potentially usable by-products (C _1_ and C _2_)

**Figure 4.**
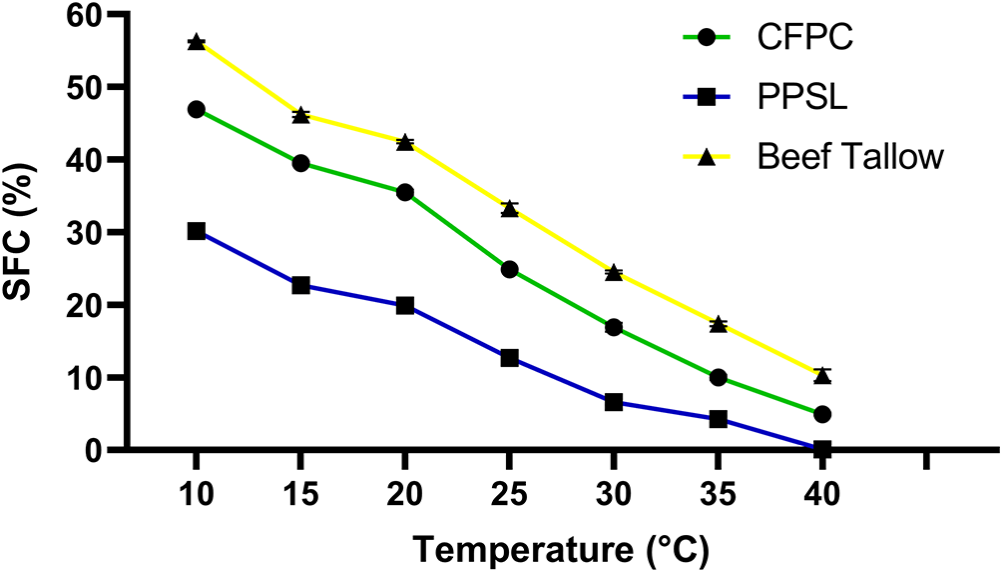
Solid fat content as a function of temperature.

Figure 4 shows the solid fat content as a function of temperature for by-products C _1_ and C _2_, and a sample of refined commercial beef tallow.

The solid fat content profile as a function of the temperature of the two by-products evaluated varied significantly with respect to a commercial beef tallow. The SFC of C _1_ and C _2_ were lower in the entire temperature range evaluated. If the SFC is analyzed together with the fatty acid profile obtained for C _1_ and C _2_, a higher percentage would be expected for C _2_, since it had a higher proportion of saturated fatty acids with a higher melting point. However, this by-product had a free acidity value three times higher than C _1_, which indicates that a greater proportion of these fatty acids were not forming a triacylglyceride. In general, beef tallow has a poor plastic range and is too firm at room temperature to meet the requirements of a “shortening”. In fact, it is usually mixed with vegetable oils or soft fats. ^19^ When analyzing the SFC profile of the C _1_ and C _2_ fatty by-products, these had a broader plastic range than beef fat, possibly due to spontaneous interesterification processes catalyzed by chemical agents, such as sodium sulfide, potassium hydroxide and lime, used in the process of tanning, unhairing, conservation and preparation. The SFC profile obtained for C _1_ and C _2_ was similar to some reported in the literature for hard and fluid “shortenings”. ^20^ However, the peroxide index values for both by-products exceed the typical values, 2 mEq O _2_/kg, for this type of products obtained by interesterification. ^21^

## Conclusion

The by-products obtained from the gelatin production process were analyzed, two of them obtained in the extraction stage were identified as potentially usable due to their high fat content. Once identified and analyzed, it was determined that they did not comply with at least one of the parameters established in resolution 2154 that states the physicochemical requirements for food tallow. The rheological and thermal characteristics, the fatty acid profile and the solid fat content showed that the previous treatments and those carried out in the preparation stages affect the properties with respect to a commercial beef fat. The solid fat content as a function of the temperature of the by-products showed a wider plastic range than commercial beef fat, however, the peroxide and acidity index limit its spectrum of applicability. These by-products could be valued and used in the formulation of animal feed, however, for applications such as biodiesel production or the food industry, they present some limitations. Then, it is important to evaluate some alternatives such as thermal fractionation to obtain fractions with a broader potential application or the reduction of free fatty acids for their potential use for biodiesel production.

## Ethics statement

Not required.

## Data Availability

Figshare: https://doi.org/10.6084/m9.figshare.21603474. ^22^ This project contains the following underlying data: Table 1. Raw data - Physicochemical parameters. Table 2. Raw data - Alteration and oxidation parameters Table 3. Raw data - Sample C1 (CFPC) FAMEs CG MS Table 3. Raw data - Sample C2 (PPSL) - FAMEs CG MS Table 4 and Figure 3. Raw data - Thermal behavior DSC C1 and C2 Figure 2. Raw data - Rheological profile C1 (CFPC) and C2 (PPSL) Figure 4. Raw data - RMN Solid Fat Content Data are available under the terms of the Creative Commons Zero “No rights reserved” data waiver (CC0 1.0 Public domain dedication).
