# Characterization of By-products with High Fat Content Derived from the Production of Bovine Gelatin

**DOI:** 10.12688/f1000research.128622.2

**Published:** 2023-09-05

**Authors:** Victor Alonso Garcia Londoño, Natalia Marín González, Diego Fernando Roa-Acosta, Lina Marcela Agudelo Laverde, Laura Botero, Liliana Maria Lellesch

**Affiliations:** 1 Departamento de Química Orgánica FCEN, Universidad de Buenos Aires, Ciudad Autónoma de Buenos Aires, Buenos Aires, 1428, Argentina; 2 Instituto de Tecnología en Polímeros y Nanotecnología, UBA-CONICET, Ciudad Autónoma de Buenos Aires, Buenos Aires, 1128, Argentina; 3 Laboratorio de Investigación, Desarrollo e Innovación, PROGEL S.A.S, Manizales, Caldas, 170001, Colombia; 4 Departamento de Agroindustria, Universidad del Cauca, Popayán, Cauca, 190001, Colombia; 5 Programa de Ingeniería de Alimentos, Universidad del Quindío, Armenia, Quíndio, 630001, Colombia

**Keywords:** fats, gelatin, by-products, characterization, circular economy

## Abstract

**Background:** Gelatin is a protein obtained by partial hydrolysis of collagen contained in skins, connective tissue and/or animal bones, which are by-products of the meat industry. The main raw material to produce bovine gelatin is the dermis of the skin, but there is a variation in fat and moisture content depending on the bovine skin origin. As a contribution to the circular economy and sustainability, these by-products with high fat content and the fat released from them during the gelatin production process can be managed for food industries, mainly in the development or formulation of animal feed.

**Methods:** For the initial physicochemical characterization, moisture, fat, protein and ashes content were determined. Once the by-products with high fat content were identified, alteration parameters such as acidity, peroxide and saponification indexes were evaluated. Additionally, thermal, rheological and fatty acid composition characterization was carried out in order to study the possible applications of the by-products.

**Results and Discussion:** The results showed that certain by-products have a fat content of less than 15%, so the viability of their use is limited. On the other hand, some by-products have a fat content exceeding 30%; however, their extraction can only be done manually, resulting in a low efficiency process. By-products removed from the supernatant in the extractors presented fat percentages of 99.9 and 98.9%, and there exists the possibility of implementing a mechanical method for their extraction. The analysis of alteration and oxidation parameters, thermal and rheological characterization, fatty acid profile and solid fat content were exclusively conducted on these high-fat content by-products. Based on the characterization, these by-products could be valued and incorporated into animal feed formulations. Nevertheless, certain limitations exist for their use in applications such as biodiesel production or the food industry.

## Introduction

The gelatin production process comprises three main steps: preparation, processing and mixing. The preparation step involves getting the raw material ready for processing in the production area. It begins with the reception of the cattle hides, which undergo basic and acid treatments to facilitate collagen extraction. During the processing step, collagen extraction and concentration take place. The concentrated “liquor” goes through a high temperature treatment (sterilization) to eliminate any possible microorganisms present. The sterilized “liquor” is then chilled and extruded (in cold) as noodles, and the resulting gelled material is deposited as a bed onto an endless open-weave stainless steel belt, and subsequently dehydrated in a drying tunnel. Finally, the dried gelatin is crushed and ground. In the mixing step, the ground gelatin is physiochemically analyzed and blended according to the specific requirements of each client. In the gelatin production process, bovine raw materials can be of national origin (CPFC) or imported (PPSL), and used in five different forms: dry, whole, leather, selvedge and trimmings (the latter correspond to different parts of the animal which include udders, gonads, prepuces, and faces, having the highest percentage of fat). The dermis of bovine leather is a by-product of the meat industry and contains collagen, molecule of interest in the gelatin production process, within its native structure. It is worth mentioning, in order to achieve the recovery of gelatin extracted from solid leather waste and contribute to the circular economy, innovative applications have been developed where gelatin can be used, for example, as a starch-based biomass adsorbent for treating discharged wastewater ^1^ or as part of hydrogel films for antibacterial wound dressing. ^2^

As mentioned, the production of gelatin takes advantage of by-products generated in the leather and meat industry. However, in the literature review, no studies were found concerning the use of by-products generated in the gelatin production process. The non-use or under-use of this by-products not only leads to potential income loss, but also represents an increase in the cost for their elimination or disposal. ^3^ Traditional uses for protein-rich by-products include food, pet food, livestock feed, and fertilizers. With regard to fatty by-products of animal origin, different alternatives have been proposed for their use and recovery in recent years: chemical transformation into soaps (derived from fatty acids), formulation of products for the food industry or reintroduction into the food chain as balanced feed for animals, after undergoing physical and chemical treatment. ^4^ Recently, new alternative uses of fat as energy/fuel sources have been developed, such as the production of biodiesel. ^5^ Any raw material that contains triacyl glycerides can serve as suitable sources for biodiesel production, including sunflower oil, rapeseed oil, soybean oil, used frying oils, lard and beef tallow. While some countries primarily produce biofuels from oils extracted from oilseeds, particularly sunflower (in Spain and Italy) and rapeseed (in Central European countries), there has been a rise in the popularization of using waste from used vegetable oils and animal fats. ^6^ ^–^ ^8^ Different products have been formulated from beef fat for the food industry, including substitutes for cocoa butter, ^9^ fat bases for the production of margarine with plastic characteristics, ^10^ shortenings ^11^ and products for the baking industry. ^8^ In some cases, the fatty acid composition of the by-product remains similar to the original product, but in other cases, significant variability can be observed. These differences can also extend to other factors or properties that impact on nutritional value, stability or overall quality. ^12^

The characterization of by-products is essential since, for example, it enables the prediction of fat behavior during its processing for biofuel production or its potential applications in the food industry. Commonly evaluated parameters include acidity, iodine and saponification indexes, humidity, fatty acid profile and triacyl glycerides content. ^7^ Another important aspect when characterizing these by-products is the evaluation of possible alterations. A typical alteration is the autoxidation of lipids, a primarily cause of the deterioration of fat-rich foods, leading to the development of unpleasant odors and flavors. This oxidation primarily occurs with unsaturated fatty acids through a series of free radical chain reactions. Another deterioration reaction is lipolysis, which results from the hydrolysis of lipid ester bonds (enzymatic by the action of lipases or by heat in the presence of water). In animal fats, lipolysis releases fatty acids, including short-chain ones that are responsible for off-flavors and off-odors. To determine the extent of oxidation development, several chemical indices are commonly used: peroxide index (PI), which measures the number of peroxides formed; thiobarbituric acid reactive substances (TBARS), which quantify the secondary products of lipid oxidation; and acidity index that determines the degree of hydrolysis of an oil or fat. ^12^ Understanding the functions and properties of fats and oil bases is essential for designing potential applications and uses or achieving products with the desired final attributes. The satisfactory performance of fats depend on critical elements that determine its applicability: stability during the post-processing period, total compatibility with the intended product and physical and functional characteristics, such as plasticity and spreadability. ^13^ Therefore, an application study should primarily be based on understanding the relationships among several parameters, such as fatty acid and triacylglyceride composition, melting point, solid fat content, thermal behavior, crystallization, microstructure and rheological properties. ^14^

The objectives of the present study were to carry out the physicochemical characterization of the by-products generated in the gelatin production process, and to analyze various parameters, including oxidation and alteration, thermal behavior, rheological profile, fatty acid profile and solid fat content of the potentially usable fatty by-products.

## Methods

### Sampling

**Figure 1.**
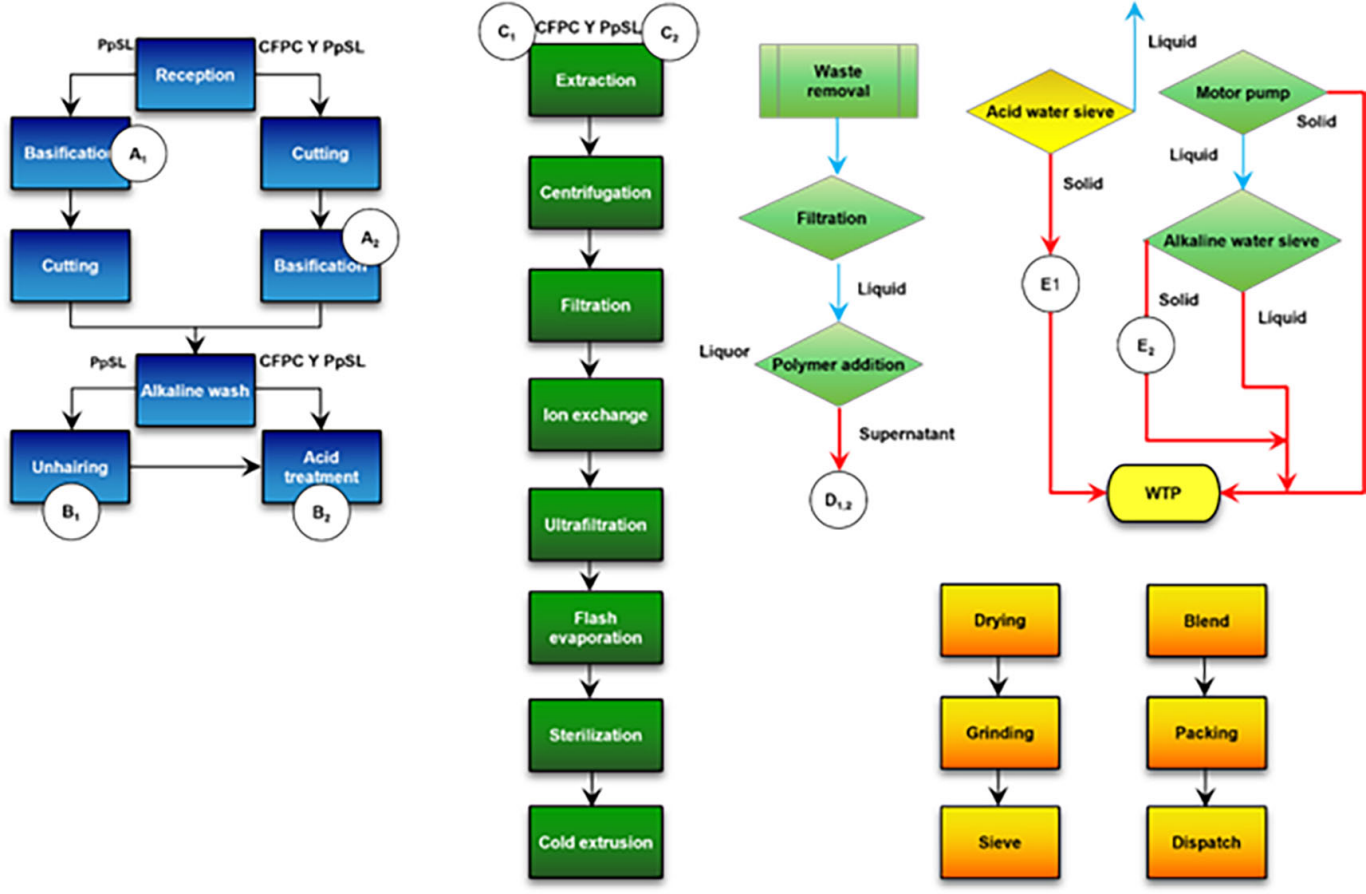
Flow chart and sampling points for fatty by-products.

The sampling of the by-products was carried out in the preparation, processing and wastewater treatment plant (WTP) areas. A total of 10 points were sampled, differentiating by raw material in those points where CFPC and PPSL were treated independently (basification, alkaline wash, and extraction). Figure 1 shows the flow chart and the sampling points.

Sampling was conducted over three consecutive days (29/08/2022 to 31/08/2022) and each sample was analyzed in triplicate for all tests.

### Physicochemical identification of potentially usable by-products

***Moisture content determination***

Moisture content was determined by the indirect method AOAC (Association of Official Analytical Chemists) 964.22, ^15^ where 5±0.005 g of sample was weighed and dried at 110°C in a forced air oven until constant weight. Constant weight was achieved after approximately 16 h of drying. Weight loss was considered as moisture content and dry residue was considered as dry matter. The results were expressed as a percentage.

***Fat content determination***

Fat content was determined by the direct organic solvent extraction method, AOAC, 960.39. ^15^ Due to the high moisture content of the by-products, the measurement was performed on the dry matter obtained after moisture determination. The dehydrated material was quantitatively transferred to a cellulose cartridge, covered with cotton and placed in the Soxhlet extractor. Extraction was carried out with anhydrous ethyl ether for 4 h, with heating at a condensation rate of 5 or 6 drops per second. The extracted solution was then quantitatively transferred to an Erlenmeyer flask and the remaining solvent was evaporated in a water bath and dried at 100°C for 30 min. Finally, the sample was cooled and weighed. The results were expressed as a percentage of fat on wet basis.

***Protein content determination***

Protein determination was performed by the Kjeldahl-Arnold-Gunning method (AOAC, 928.08), ^15^ by digesting 0.8 ± 0.01 g of sample in a Kjeldahl digestion tube with 6 g of Na _2_SO _4_, 0.3 g of CuSO _4_ and 12 ml of concentrated H _2_SO _4_. The digestion was carried out in a Selecta digester (PRO-NITRO M) under the following conditions: 125°C – 15 minutes; 300°C – 15 minutes; 400°C – 90 minutes. The distillation was carried out in a Selecta automatic Kjeldahl Distiller (Pro-nitro). The digested sample was alkalized and distilled by steam addition. The distillate was collected in 50 ml of saturated boric acid solution with combined indicator. The nitrogen content was determined by titration with 0.1N HCl. Results were expressed as protein percentage using a conversion factor of 6.25.

***Ash content determination***

Based on AOAC 923.03 ^15^ method, 5 ± 0.01 g of sample was placed in a porcelain capsule of 6 cm of diameter. The sample was first incinerated with a Bunsen burner until complete carbonization and then in a high-temperature muffle furnace at 500°—550°C until constant weight. Finally, it was cooled in a desiccator and weighed. Results were expressed as ash percentage.

### Characterization of potentially usable fatty by-products (alteration and oxidation index, and thermal and rheological characteristics, fatty acid profile and solid fat content)

***Saponification index (SI)***

According to the CTS (Colombian Technical Standard) 335, ^16^ 2 ± 0.005 g of sample was weighed in an Erlenmeyer flask. Then, 25 mL of an ethanolic solution of potassium hydroxide, along with boiling aids, were added to the flask. The reflux condenser was connected to the flask and the solution was allowed to boil slightly while being stirred sporadically. After 60 min of boiling, the condenser was removed and 0.5 mL of phenolphthalein solution was added to the hot solution. It was then titrated with 0.1 N HCl. A reagent blank was analyzed in the same way. The results were expressed as the number of milligrams of KOH required to saponify 1 g of fat.

***Acidity index (AI)***

The acidity index was performed following the procedure established in the CTS 218. ^17^ A sample of 2.5 ± 0.005 g was weighed in a flask. In a separate flask, 50 mL of ethanol containing 0.5 mL of phenolphthalein was heated to boiling. While the temperature was above 70°C, it was neutralized using a 0.1 N KOH solution. The neutralized ethanol was then added to the flask with the sample and the mixture was mixed and boiled. Finally, it was titrated with 0.1 N KOH solution. The results were expressed as the number of milligrams of KOH required to neutralize free fatty acids in 1 g of sample.

***Peroxide index (PI)***

According to the Colombian Technical Standard 236, ^18^ 5.0 ± 0.1 g of sample was weighed and 30 mL of acetic/chloroform solution (3:2) was added. The sample was dissolved by shaking and 0.5 ml of KI solution was added. The flask was covered and stirred and then left to rest for 60 s before adding 30 mL of water. The released iodine was immediately titrated with 0.01 N sodium thiosulfate solution until the solution turned pale-yellow in color. Then, 0.5 mL of starch solution was added and the titration was continued until the solution became colorless. A reagent blank was also analyzed. The results were expressed in milliequivalents of active oxygen per kg of sample.

***Determination of fatty acid methyl esters (FAMEs)***

For the determination of fatty acid methyl esters, a weighed sample (0.7 g) was placed in a 50 mL three-necked flask with 6 mL of a 0.5 M NaOH solution. The flask was placed at reflux, with the temperature controlled at a maximum of 60°C, until the sample was completely dissolved. Twenty minutes were counted from that moment. Afterward, the sample was derivatized with 5 mL of 14% BF3 in MeOH and the reaction was continued for other 20 minutes. Finally, 3 mL of n-heptane was added. Once cooled, the derivatized sample was taken to a test tube and 15 mL of saturated NaCl solution was added. An aliquot of 1.5 mL was taken of the organic phase and placed in a vial for further gas chromatography analysis.

Gas chromatography analysis was conducted using a Shimadzu GC-MS QP-2010 mass spectrometer, equipped with an AOC-20i autoinjector and an AOC-20s autosampler. Chromatographic conditions were: Zebron ZB-5 column 30 m × 0.25 mm I. D, 0.25 μm, temperature ramp programmed from 100 to 300°C (2 min of initial heating at 100°C, followed by a temperature was increase of 7°C/min up to 300°C, which was held for 5 min). The temperature at the injection port was 250°C and the injection was in split mode (1:20). The column flow was 1.00 ml/min, the total flow rate was 24.0 ml/min. The ion source and interface temperatures were maintained at 290°C. The total analysis time was 36 min.

### Rheological profile

An AR 1500 rheometer (TA Instruments, New Castel, USA) equipped with a Peltier temperature control system in the lower plate. The upper plate had a diameter of 40 mm and a 0° angle. A gap of 1.5 mm was set between the lower and upper plates. Before the measurements, the sample was conditioned for 2 min at the desire measurement temperature. A shear stress (Pa) scan was performed as a function of time (5 min) at a shear rate of 50 (s ^-1^). The obtained data were modeled by the Weltman equation, where the parameter A is the instantaneous effort necessary to start the structure de-structuring process during shearing and parameter B indicates the rate of de-structuring of the fats. ^13^ The rheological profile was performed at 25°C and 35°C.

$$\text{Effort}=A-B*\mathrm{Ln}\;\left(\text{time}\right)$$

### Thermal behavior

Thermal behavior was determined by differential scanning calorimetry (DSC) with a Netzsch polyma 214 calorimeter. A gaseous N _2_ flow at 50 mL/min was used as carrier gas to avoid humidity in the measurement cell. About 9 mg of each sample was weighed into hermetically sealed 40 μL aluminum pans. The samples were melted at 80°C (heating speed of 10°C/min) for 15 min. Subsequently, cooling was carried out at 10°C/min until reaching 0°C, and the samples were held at this temperature for 30 min. Then, the samples were heated again up to 20°C (10°C/min) and held at this temperature for 30 min. Finally, they were once again melted at 80°C (heating rate of 5°C/min) for 2 min.

### Solid fat content as a function of temperature

Solid fat content (SFC) of the samples at equilibrium was determined with a Bruker mq 20 Minispec equipment that has a cell with temperature control and a magnetic field of 0.47 Tesla, which operates at a frequency of 20 MHz. A 10 mm diameter glass tube specific for NMR, with approximately 6 mL of sample, was used. Solid fat content (SFC) was studied following the methodology described in the American Oil Chemists' Society (AOCS Cd 16b 93 for confectionery fats). ^19^ The samples were prepared in triplicate and the results were expressed by the average of three values and their standard deviation. The experimental protocol was: the sample was first melted at 100°C and held isothermally for 15 min in order to erase its thermal history, then it was cooled to 60°C for 5 min, followed by further cooling to 0°C for 90 min. The sample was then tempered at 26°C for a precise duration of 40±0.5 hours. Subsequently, it underwent another cooling step at 0°C for 90 minutes. Finally, it was kept for 60 min at each of the selected crystallization temperatures (CT) for the determination of the SFC of the fat from extractors (CFPC and PPSL): 10, 15, 20, 25, 30, 35 and 40°C.

### Statistical analysis

Analysis of variance (ANOVA) was performed using the statistical program Statgraphics Centurion version XVI (Statistical graphics Corporation, USA), comparing the means by the least significant difference test of Fisher (LSD) with a confidence level of 95.

## Results and discusion

### Physicochemical identification of potentially usable by-products

The physicochemical parameters of the identified fatty by-products are shown in Table 1.

**Table 1.** Physicochemical parameters of the identified fatty by-products (n.d: not determined).

Area	Sampling location	Raw material	Moisture (%)	Fat (%)	Protein (%)	Ash (%)
Preparation	Basification tanks	CFPC (A _1_)	42.2±0.8	39.8±3.9	6.5±0.9	1.9±0.1
		PPSL (A _2_)	48.2±16	30.7±1.9	3.4±1.2	2.7±0.4
	Alkaline wash	CFPC (B _1_)	74.6±4.4	13.6±1.4	4.9±1.2	4.1±0.4
		PPSL (B _2_)	79.9±4.8	11.7±1.7	3±0.2	4.2±0.1
Processing	Extractors	CFPC (C _1_)	n.d	99.9±0.1	n.d	0.004±0.001
		PPSL (C _2_)	n.d	98.9±0.3	n.d	0.002±0.001
	Polymer waste tank A (D _1_)	----	82.7±0.6	3.5±0.4	8.4±0.6	0.7±0.1
	Polymer waste tank B (D _2_)	----	80.5±0.6	3.7±1	8.4±0.4	0.3±0.1
WTP	Acid water sieve (E _1_)	----	87.4±2.5	7.6±0.7	6.2±0.3	2.4±0.4
	Alkaline water sieve (E _2_)	----	73.0±4.8	12±1	2±0.2	4.3±1

The results showed that certain by-products (B _1_, B _2_, D _1_, D _2_, E _1_ and E _2_) presented fat content values below 15.0%, so the viability of their use is limited. On the other hand, by-products A _1_ and A _2_ have more than 30% of fat content, however, these by-products can only be removed manually and the efficiency of this process is relatively low. By-products C _1_ and C _2_ presented 99.9 and 98.9% of fat content, respectively, and very low ash content. These fatty by-products are removed from the supernatant in the extractors, offering the possibility of implementing a mechanical method for their extraction. Consequently, the analysis of alteration and oxidation parameters, thermal and rheological characterization, fatty acid profile and solid fat content as a function of temperature was exclusively conducted on C _1_ and C _2_ by-products.

Although the primary focus of this investigation is on by-products with high fat content, it is worth mentioning that residues A _1_, A _2_, B _1_, B _2_, D _1_, and D _2_ hold potential usability due to their protein content. These residues could find applications in areas such as animal feed, provided the fat is separated beforehand.

### Alteration and oxidation parameters of potentially usable by-products (C _1_ and C _2_)

Table 2 shows the saponification, acidity and peroxide indices obtained for the potentially usable fat by-products.

**Table 2.** Alteration and oxidation parameters of potentially usable by-products.

Area	Sampling location	Raw material	SI mg KOH/g	AI mg KOH/g	PI meq O _2_/Kg
Processing	Extractors	CFPC (C _1_)	190±1	11.3±0.6	3.0±0.3
		PPSL (C _2_)	194±5	35.2±15.9	10.5±1.0

Resolution 2154 of 2012 of the Ministry of Health and Social Protection of the Republic of Colombia (chapter X) ^20^ establishes the physicochemical requirements for food tallow, defined as the product obtained by the fusion of fatty, clean and healthy tissues, including fat from trimmings and related muscles and bones, from bovine (Bos taurus) and/or sheep (Ovis aries) animals. C _1_ and C _2_ saponification index values were within the parameters established in the mentioned resolution (SI between 190 and 202 mg KOH/g), but neither of the two by-products complied with the acidity parameters (AI < 2.5 mg of KOH/g). The peroxide value for C _1_ was within the established limit (<10 meq active oxygen/kg), whereas C _2_ was just within the specified limit. The difference in the oxidation and alteration parameters between the fatty by-products obtained from the two raw materials, CFPC (C _1_) and PPSL (C _2_), can be attributed to the chemical treatments and transport times they undergo before arriving to the gelatin factory. CFPC is unhaired in local tanneries where sulfides are generally used. Normally, this material arrives at the gelatin factory between 2 and 4 days after being processed in the tannery. PPSL is a raw material imported principally from Argentina and the United States, and for its conservation during maritime transport, salt or brines are added. Moreover, the minimum time between its obtention and its arrival to the gelatin factory in Colombia is 15 days, time enough to leave oxidation and alteration processes to occur. Although neither of the two by-products complied with the parameters of the resolution 2154, ^20^ there are potential strategies for reducing free fatty acids ^21^ ^,^ ^22^ that could be evaluated in the future.

### Determination of fatty acid methyl esters (FAMEs) of potentially usable by-products (C _1_ and C _2_)

Table 3 shows the percentage of fatty acids for C _1_, C _2_ and a commercial refined beef tallow.

**Table 3.** Percentage of fatty acid methyl esters of a commercial beef tallow, C _1_ and C _2_.

Retention time	FAME	Name	% Composition		
			Beef tallow	CFPC (C _1_)	PPSL (C _2_)
9.8	C14:0	Myristic acid	3.1	3.7	3.4
12.6	C16:0	Palmitic acid	25.7	27.8	34.1
13.12	C16:1	Palmitoleic acid	2.9	3.7	0.5
16.66	C18:0	Stearic acid	22.0	18.3	21.7
17.37	C18:1	Oleic acid	39.2	37.7	37.9
18.26	C18:2	Linoleic acid	2.3	2.8	0.0
18.93	C18:3	Linolenic acid	0.6	0.0	0.0
21.48	C20:0	Arachidonic acid	0.1	1.1	0.5
22.06	C20:1	Eicosenoic acid	0.4	0.0	0.0
		**Saturated**	51.0	50.9	59.6
		**Insaturated**	46.0	44.2	38.4

The fatty acid profile obtained for C _1_ and C _2_ was similar to that of beef tallow. However, it was observed that C _2_ had a lower percentage of unsaturated fatty acids, possibly due to autoxidation or rancidity processes generated by the previous treatment in tanneries and during the storage and transport period. Beef tallow is a raw material commonly used in the biodiesel industry, but it has unfavorable properties due to its high concentration of saturated fatty esters (stearic and palmitic). In fact, to overcome this limitation it is often blended with other raw materials that have higher content of unsaturated fatty acids. ^23^ Considering that C _2_ has approximately 9% more saturated fatty acids than standard beef tallow and a high content of free fatty acids, this by-product may not be suitable for the biodiesel industry. Nevertheless, there are alternative approaches to utilize these by-products, such as obtaining fractions with different properties through thermal fractionation ^24^ or chemical or enzymatic esterification methods. ^25^

### Rheological profile of potentially usable by-products (C _1_ and C _2_)

**Figure 2.**
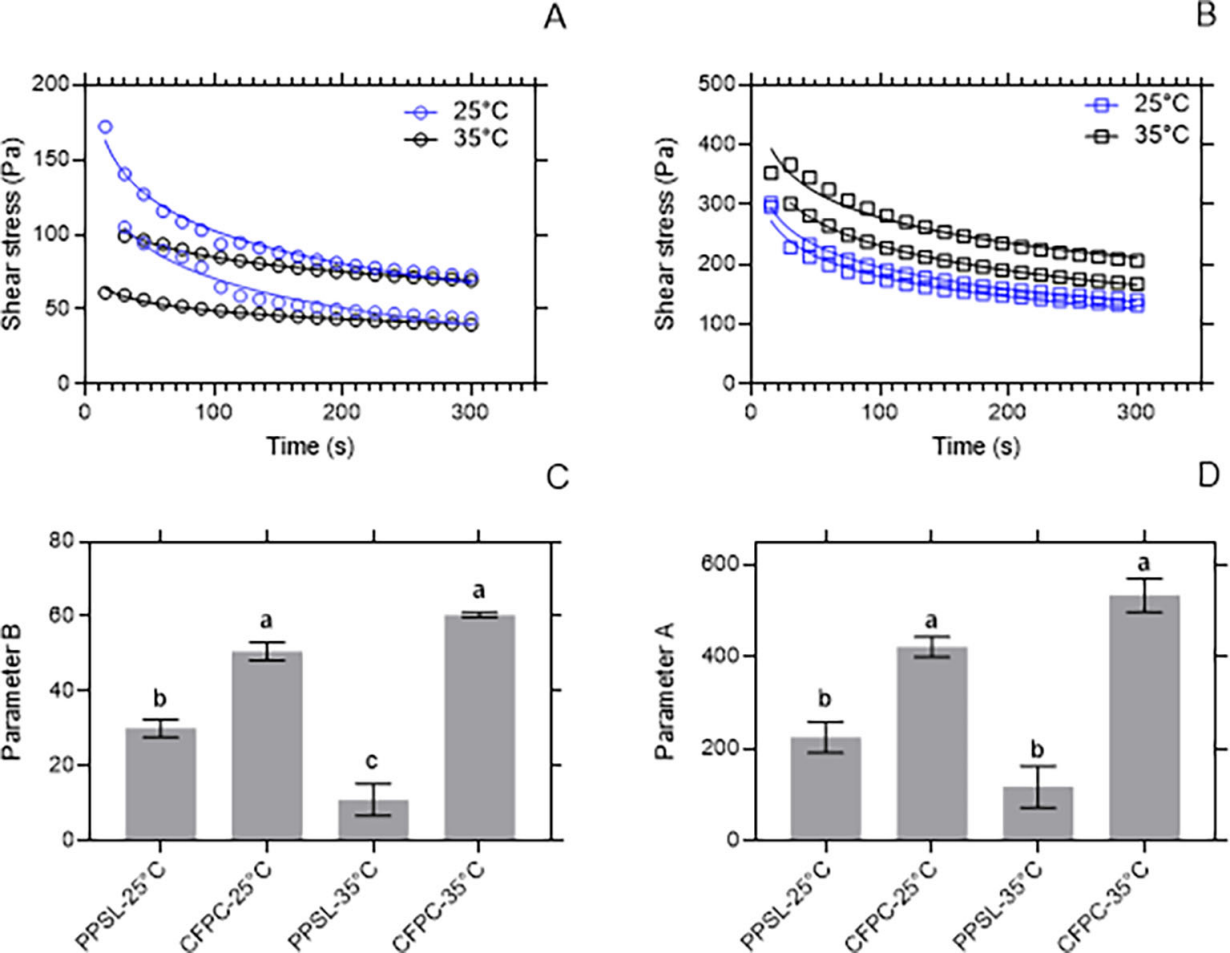
Rheological profile of potentially usable by-products. (A) shows the shear stress behavior for C _2_ subjected to a shear rate of 50 s ^-1^ for 5 minutes at 25°C and 35°C. (B) shows the shear stress behavior for C _1_ subjected to a shear rate of 50 s ^-1^ for 5 minutes at 25°C and 35°C. (C) shows the rate of de-structuring (parameter B) for C _1_ and C _2_ at 25°C and 35°C. (D) shows the initial resistance (parameter A) for C _1_ and C _2_ at 25°C and 35°C.

To optimize the potential of animal fat waste as food ingredient or biodiesel feedstock on a large scale, it is crucial to analyze the thermal and rheological behavior of these materials to guide the design of systems and the biodiesel production process. ^26^ Figures 2A and 2B show the decrease of the shear stress of C _1_ and C _2_ with time. This behavior was also found by Adewale et al. ^26^ for beef tallow and is characteristic of thixotropy, where the shear stress tends to become constant with time until reaching an equilibrium shear stress (σe). By-product C _1_ (CFPC) showed σe greater than 150 Pa, while the σe of C _2_ was less than 100 Pa. The parameter B of C _1_ was greater than that of C _2_. This could indicate that C _1_ destructures faster under shear stress, so it can slide more easily over a surface than C _2_. It is important to note that this ease of sliding on a surface does not change significantly with an increase of 10°C of temperature. Otherwise, parameter B of C _2_ is affected by the temperature change ( Figure 2C). It is also observed that the initial resistance factor (parameter A) was higher in C _1_ and this did not show significant dependence on the temperature. This behavior is influenced by the composition of saturated and unsaturated fatty acids in each sample.

By-product C _1_ could be suitable for use in the preparation of puff pastry-type bakery products, in which fats that can easily mix with flour are needed.

### Thermal behavior of potentially usable by-products (C _1_ and C _2_)

**Figure 3.**
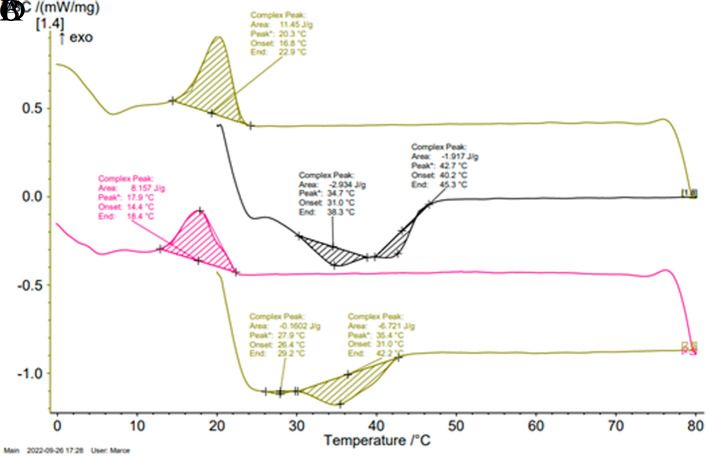
Typical thermograms obtained for by-products C _1_ and C _2_. (A) Shows the crystallization peak of C _1_. (B) Shows the melting peaks of C _1_. (C) Shows the crystallization peak at 20°C of C _2_. (D) Shows the melting peaks of C _2_.

The thermograms showed in Figures 3A and 3C exhibit the melting and crystallization curves of the fatty by-products determined by differential scanning calorimetry. During cooling, one exothermic peak -attributed to the phenomenon of fat crystallization was observed in each sample whereas, upon heating, two endothermic peaks were observed in their thermograms. The presence of multiple endothermic peaks in the samples could arise from a solid–solid transition occurring between two distinct crystalline phases, or alternatively, it could result from the melting of various crystalline phases; due the polymorphism of animal fat. ^26^

The onset of the crystallization phenomenon in the C _2_ by-product (14.4°C) occurred at slightly lower temperatures than in C _1_ (16.8°C); however, no significant differences were observed in the crystallization temperature between the two by-products (17.9-20.3°C). These peak temperatures were comparatively lower in relation to the values reported in literature for beef tallow (23-29°C). ^26^ ^,^ ^27^ Both by-products thermograms showed an endothermic peak at 35°C, however, in the C _2_ thermogram, there was a peak at 28°C absent in that of C _1_. On the other hand, C _1_ showed a peak at 43°C that was not evidenced in C _2_ thermogram. These results were correlated with the behavior in the solid fat percentage profiles as a function of temperature. Applying thermal fractionation to the C _1_ by-product would yield two fractions with different applicability range, enhancing its value.

### Solid fat content as a function of temperature (SFC) of potentially usable by-products (C _1_ and C _2_)

**Figure 4.**
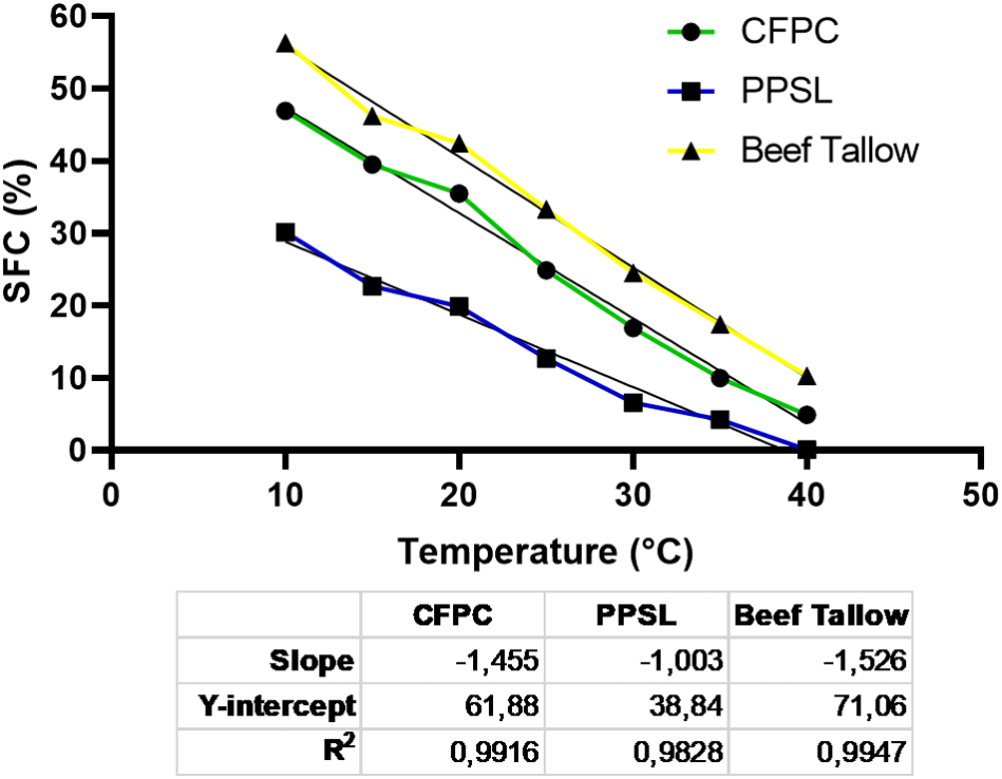
Solid fat content as a function of temperature.

Figure 4 shows the solid fat content as a function of temperature for C _1_ and C _2_ by-products, and a sample of refined commercial beef tallow.

**Table 4.** Temperatures and areas of crystallization and melting determined in the by-products C _1_ and C _2_ by DSC.

By-product	Crystallization		Melting			
	Area J/g	Tp °C	Area J/g	Tp °C	Area J/g	Tp °C
**C**_**1**_ **(CFPC)**	10.8±1.1	21.4±1.1 ^a^	1.8±0.2	34.9±0.1 ^c^	3.4±0.7	43±0.3 ^e^
**C**_**2**_ **(PPSL)**	6.9±1.9	18.8±2.7 ^a^	0.3±0.2	28.1±0.5 ^d^	5.7±1.3	35.5±0.2 ^c^

Results are mean of three determinations±standard deviation.

The means with different letters in the superscript are significantly different (p<0.05).

The solid fat content profile as a function of the temperature of the two evaluated by-products varied significantly with respect to a commercial beef tallow. The SFC of C _1_ and C _2_ were lower in the entire temperature range evaluated. If the SFC is analyzed together with the fatty acid profile obtained for C _1_ and C _2_, a higher percentage would be expected for C _2_, since it had a higher proportion of saturated fatty acids with a higher melting point. However, this by-product had a free acidity value three times higher than C _1_, which indicates that a greater proportion of these fatty acids were not forming a triacylglyceride. In general, beef tallow has a poor plastic range and is too firm at room temperature to meet the requirements of a “shortening”. In fact, it is usually blended with vegetable oils or soft fats. ^28^ When analyzing the SFC profile of the C _1_ and C _2_ fatty by-products, it was observed that they had a broader plastic range compared to beef fat. This broader range could be attributed to spontaneous interesterification processes catalyzed by chemical agents, such as sodium sulfide, potassium hydroxide and lime, used in the process of tanning, unhairing, conservation and preparation. The SFC profile obtained for C _1_ and C _2_ was similar to some reported in the literature for hard and fluid “shortenings”. ^29^ However, the peroxide index values for both by-products exceeded the typical values (2 mEq O _2_/kg) for this type of products obtained by interesterification. ^30^

## Conclusion

The by-products obtained from the gelatin production process were analyzed, and two of them obtained in the extraction stage were identified as potentially usable due to their high fat content. Upon analysis, it was determined that they did not meet at least one of the parameters established in resolution 2154 which states the physicochemical requirements for food tallow. The rheological and thermal characteristics, fatty acid profile and solid fat content revealed that the previous treatments and those carried out in the preparation stages affected the properties in comparison to commercial beef fat. The solid fat content of the by-products showed a wider plastic range than commercial beef fat, however, the peroxide and acidity index confer some limitations on their applicability spectrum. While these by-products could find value in the formulation of animal feed, they have certain constraints for applications in biodiesel production and the food industry. Then, it is important to explore alternative methods such as thermal fractionation, to obtain fractions with broader potential applications, or the reduction of free fatty acids to enhance their suitability for biodiesel production.

## Ethics statement

Not required.

## Data Availability

Figshare: https://doi.org/10.6084/m9.figshare.21603474. ^32^ This project contains the following underlying data: Table 1. Raw data - Physicochemical parameters. Table 2. Raw data - Alteration and oxidation parameters Table 3. Raw data - Sample C1 (CFPC) FAMEs CG MS Table 3. Raw data - Sample C2 (PPSL) - FAMEs CG MS Table 4 and Figure 3. Raw data - Thermal behavior DSC C1 and C2 Figure 2. Raw data - Rheological profile C1 (CFPC) and C2 (PPSL) Figure 4. Raw data - RMN Solid Fat Content Data are available under the terms of the Creative Commons Zero “No rights reserved” data waiver (CC0 1.0 Public domain dedication).
