# Supplementary material for: Characterization of By-products with High Fat Content Derived from the Production of Bovine Gelatin
Source: F1000Res. 2023 Sep 5;11:1575. Originally published 2022 Dec 23. [Version 2] doi: 10.12688/f1000research.128622.2 (PMC10511850; doi:10.12688/f1000research.128622.2)
Supplement: Peer review history

# Reviewer report: Reviewer response for version 2

**DOI:** 10.5256/f1000research.154471.r204208

**Published:** 2023-09-20

**Authors:** Xugang Dang

**Affiliations:** 1 College of Bioresources Chemistry and Materials Engineering, Shaanxi University of Science and Technology, Xi'an, Shaanxi, China

The revised paper can be indexed.

Is the work clearly and accurately presented and does it cite the current literature?

Partly

If applicable, is the statistical analysis and its interpretation appropriate?

Partly

Are all the source data underlying the results available to ensure full reproducibility?

Partly

Is the study design appropriate and is the work technically sound?

Yes

Are the conclusions drawn adequately supported by the results?

Yes

Are sufficient details of methods and analysis provided to allow replication by others?

Partly

Reviewer Expertise:

Natural biomass and biopolymers

I confirm that I have read this submission and believe that I have an appropriate level of expertise to confirm that it is of an acceptable scientific standard.

# Reviewer report: Reviewer response for version 1

**DOI:** 10.5256/f1000research.141232.r173259

**Published:** 2023-05-26

**Authors:** Xugang Dang

**Affiliations:** 1 College of Bioresources Chemistry and Materials Engineering, Shaanxi University of Science and Technology, Xi'an, Shaanxi, China

1. Abstract and throughout the manuscript: the authors should present more tangible data (values, %, etc.).
2. The introduction section should be reasonably organized, highlighting key innovations and discoveries. Partial paragraphs should be merged.
3. The references should be updated to recent years.
4. The authors mentioned that the gelatin has a wide range of applications because of their excellent properties. The related references should be cited: Zhang *et al.* (2021 ^1^) and Dang *et al.* (2022 ^2^).
5. The results and discussion section should add the related references to compare and discuss the key innovations and discoveries of the research.
6. The English of the whole manuscript should be improved.
7. The results section should be revised to results and discussion.

Is the work clearly and accurately presented and does it cite the current literature?

Partly

If applicable, is the statistical analysis and its interpretation appropriate?

Partly

Are all the source data underlying the results available to ensure full reproducibility?

Partly

Is the study design appropriate and is the work technically sound?

Yes

Are the conclusions drawn adequately supported by the results?

Yes

Are sufficient details of methods and analysis provided to allow replication by others?

Partly

Reviewer Expertise:

Natural biomass and biopolymers

I confirm that I have read this submission and believe that I have an appropriate level of expertise to confirm that it is of an acceptable scientific standard, however I have significant reservations, as outlined above.

## References

[1] : High-mechanical strength carboxymethyl chitosan-based hydrogel film for antibacterial wound dressing. *Carbohydr Polym* .2021;256: 10.1016/j.carbpol.2020.117590 117590 10.1016/j.carbpol.2020.117590 33483076

[2] : Sustainable electrochemical synthesis of natural starch-based biomass adsorbent with ultrahigh adsorption capacity for Cr(VI) and dyes removal. *Separation and Purification Technology* .2022;288: 10.1016/j.seppur.2022.120668 10.1016/j.seppur.2022.120668

# Response

**Published:** 2023-08-10

**Authors:** Victor Alonso Garcia Londoño

**Affiliations:** Instituto de Tecnología en Polímeros y Nanotecnología UBA-CONICET, Argentina

Comments to Reviewer 2:

  

- **Abstract and throughout the manuscript: the authors should present more tangible data (values, %, etc.).**

Abstract was written according the journal author guide. More data was throughout the manuscript.

- **The introduction section should be reasonably organized, highlighting key innovations and discoveries. Partial paragraphs should be merged.**

Introduction section was improved: English writing was revised; partial paragraphs were merged and some cites were added.

- **The references should be updated to recent years**.

Some updated references were added throughout the manuscript. Nevertheless, it is important to consider that no papers were found on fatty by-products in the gelatin industry.

- **The authors mentioned that the gelatin has a wide range of applications because of their excellent properties. The related references should be cited: Zhang *et al.* (2021 ^1^ ) and Dang *et al.* (2022 ^2^ ).**

The following paragraph has been added to the introduction, where both suggested references were added: “It is worth mentioning, in order to achieve the recovery of gelatin extracted from solid leather waste and contribute to the circular economy, innovative applications have been developed where gelatin can be used, for example, as a starch-based biomass adsorbent for treating discharged wastewater ^1^ or as part of hydrogel films for antibacterial wound dressing ^2^.”

- **The results and discussion section should add the related references to compare and discuss the key innovations and discoveries of the research.**

Some related references were added throughout the Results and Discussion section. However, it is important to consider that no papers were found on fatty by-products in the gelatin industry.

- **The English of the whole manuscript should be improved.**

The manuscript has been subject to revision and improvement in its English writing.

  

- **The results section should be revised to results and discussion.**

The discussion was included in the Results section. Anyway, the title of the section was changed to “Results and discussion”.

 A new version of the revised manuscript was submitted.

# Reviewer report: Reviewer response for version 1

**DOI:** 10.5256/f1000research.141232.r159201

**Published:** 2023-01-19

**Authors:** Julián Andres Gómez Salazar

**Affiliations:** 1 Food Department, University of Guanajuato, Irapuato, Mexico

General comment: The authors studied the physicochemical characterization of by-products derived from the production of bovine gelatin. They determined chemical (moisture, fat, protein, ashes, fatty acid) and physical parameters (thermal and rheological). This work shows the possible applications of the by-products obtained in the stages of the gelatin production process.

 The manuscript is well written and organized. The topic is interesting and has potential applications for the food and pharmaceutical industry. I suggest indexing after a minor revision. Suggestions and requirements to improve the manuscript are mentioned below:

- Indicate, what is the reason that by-products with less than 1.5% fat have low viability? (page 6). Add reference please (page 6).
- Comment on the results of the other physicochemical parameters obtained (protein, moisture, ash) (table 1) and compare them with other studies.
- Add resolution 2154 of 2012 in references (page 7).
- Please, describe the determination of the fatty acid profile of beef tallow in the methodology and add the reference. Likewise for the determination of the fat solids content in beef tallow.
- Compare the rheological results and thermal behavior with other studies carried out on similar by-products.
- Include linear correlations between fat solids content and temperature.
- Indicate the meaning of the letters in table 4. Describe the statistical analysis used.

Is the work clearly and accurately presented and does it cite the current literature?

Yes

If applicable, is the statistical analysis and its interpretation appropriate?

Yes

Are all the source data underlying the results available to ensure full reproducibility?

Yes

Is the study design appropriate and is the work technically sound?

Yes

Are the conclusions drawn adequately supported by the results?

Yes

Are sufficient details of methods and analysis provided to allow replication by others?

Yes

Reviewer Expertise:

food processing and preservation, meat technology, meat science, food chemistry

I confirm that I have read this submission and believe that I have an appropriate level of expertise to confirm that it is of an acceptable scientific standard.

# Response

**Published:** 2023-08-10

**Authors:** Victor Alonso Garcia Londoño

**Affiliations:** Instituto de Tecnología en Polímeros y Nanotecnología UBA-CONICET, Argentina

Comments to Reviewer 1:

  

- **Indicate, what is the reason that by-products with less than 1.5% fat have low viability? (page 6). Add reference please (page 6).**

Fatty by-products below 15% correspond to the alkaline wash (B1, B2), polymer waste tanks (D1 and D2), acid water sieve (E1), and alkaline water sieve (E2). Although a fat content was detected in all cases, extracting this fat would necessitate a secondary operation due to its emulsified or mixed state with protein, fiber residue, and inorganic substances like salts or minerals. This secondary operation would incur additional energy and labor costs.

 For this specific analysis, no reference is provided as it pertains exclusively to the evaluated process.

- **Comment on the results of the other physicochemical parameters obtained (protein, moisture, ash) (table 1) and compare them with other studies.**

This study represents the first-ever physical-chemical characterization conducted on by-products from the gelatin production industry. It is important to note that the primary focus of this investigation is on by-products with high fat content. However, it is worth mentioning that residues A1, A2, B1, B2, D1, and D2 hold potential usability due to their protein content. These residues could find applications in areas such as animal feed, provided the fat is separated beforehand.

 The following text was added to the manuscript: “Although the primary focus of this investigation is on by-products with high fat content, it is worth mentioning that residues A1, A2, B1, B2, D1, and D2 hold potential usability due to their protein content. These residues could find applications in areas such as animal feed, provided the fat is separated beforehand.”

- **Add resolution 2154 of 2012 in references (page 7).**

Reference added.

- **Please, describe the determination of the fatty acid profile of beef tallow in the methodology and add the reference. Likewise for the determination of the fat solids content in beef tallow.**

The fatty acid profile method is described on page 5 (heading: “Determination of fatty acid methyl esters (FAMEs)”) and the solid fat content procedure is described on page 6 (heading: “Solid fat content as a function of temperature”).

  

- **Compare the rheological results and thermal behavior with other studies carried out on similar by-products.**

Rheological and thermal results were compared with other studies. Nevertheless, it is important to consider that no papers were found on fatty by-products in the gelatin industry.

- **Include linear correlations between fat solids content and temperature.**

Linear correlations were added.

- **Indicate the meaning of the letters in table 4. Describe the statistical analysis used.**

The statistical analysis method used was added in the section Materials and Methods: “Analysis of variance (ANOVA) was performed using the statistical program Statgraphics Centurion version XVI (Statistical graphics Corporation, USA), comparing the means by the least significant difference test of Fisher (LSD) with a confidence level of 95%.”

 The following text was added to the table foot (Table 4): “Results are mean of three determinations ± standard deviation. The means with different letters in the superscript are significantly different (p <0.05).”

 A new version of the revised manuscript was submitted.
